# Transcriptomic changes in wheat during tan spot (*Pyrenophora tritici-repentis*) disease

**DOI:** 10.1186/s13104-019-4517-4

**Published:** 2019-08-01

**Authors:** Ethan J. Andersen, Shaukat Ali, Madhav P. Nepal

**Affiliations:** 1 Department of Biology and Microbiology, South Dakota State University, Brookings, SD 57007 USA; 2 Department of Agronomy, Horticulture and Plant Science, South Dakota State University, Brookings, SD 57007 USA

**Keywords:** *Pyrenophora tritici-repentis*, Wheat, Tan spot, Transcriptome, RNA-seq, Ptr ToxA

## Abstract

**Objectives:**

Tan spot is a yield-reducing disease that affects wheat and is caused by the fungus *Pyrenophora tritici-repentis* (*Ptr*). Eight races of Ptr have been identified based upon production of the effectors Ptr ToxA, Ptr ToxB, and Ptr ToxC. Wheat cultivars have also been characterized by their resistance and susceptibility to races of *Ptr* and sensitivity to the effectors. The objective of this research was to assess differences in gene expression between *Ptr* resistant and susceptible wheat cultivars when either inoculated with *Ptr* race 2 spores or directly infiltrated with Ptr ToxA.

**Data description:**

A greenhouse experiment was used to assess wheat-*Ptr* interaction. Wheat seedlings were grown for two weeks prior to the experiment under greenhouse conditions. Four treatments were used: (1) spray-inoculation with a suspension of *Ptr* spores (3000 spores/mL) (2) spray inoculation with water as a control (3) needleless syringe injection with Ptr ToxA, and (4) needleless syringe injection with water as a control. Plants were transferred to a humidity chamber and leaf sample were taken at 0, 8, and 16 h. After RNA extraction and sequencing, 48 RNA datasets are reported. This data will be useful in understanding how resistant wheat responds to *Ptr* compared to susceptible wheat.

## Objective

Tan spot is a yield-reducing disease that affects wheat and is caused by the fungus *Pyrenophora tritici-repentis* (*Ptr*) [1]. Eight races of Ptr have been identified based upon production of the effectors Ptr ToxA, Ptr ToxB, and Ptr ToxC. Races 1–8 produce the following toxins: A + C, A, C, none, B, B + C, A + B, and A + B + C, respectively [2–5]. Since Race 2 produces only Ptr ToxA, isolates of this race can be used to study the response of wheat to only Ptr ToxA. Wheat cultivars have also been characterized by their resistance and susceptibility to races of *Ptr*. The cultivar Glenlea, for example, is sensitive to Ptr ToxA but not the other toxins, whereas Salamouni is insensitive to any of the toxins [6, 7]. Sensitivity to Ptr ToxA has been linked to presence of the susceptibility gene *Tsn1* [8]. Cultivars that are insensitive to Ptr ToxA have been found to possess *Tsn1* genes with premature stop codons [8]. The objective of this research was to assess differences in gene expression between *Ptr* resistant and susceptible wheat cultivars when either inoculated with *Ptr* race 2 spores or directly infiltrated with Ptr ToxA. Understanding the differences between wheat responses triggered by the toxin versus the entire pathogen will provide insight into the mechanisms behind how wheat detects pathogens.

## Data description

### Greenhouse experiment

Wheat seedlings were grown for two weeks in 3 × 9 cm plastic cones (Stuewe & Sons Inc., Tangent, OR, USA) under greenhouse conditions (16 h light, 8 h dark, 22 °C). Both tan spot resistant Salamouni and susceptible Glenlea cultivars were grown. Ptr race 2 isolate 86–124 was grown on V8-PDA medium plates [9], incubated in darkness for five days and flooded with water to disrupt colonies, following the methods from Abdullah et al. [10]. Spore suspension at 3000 spores/mL was spayed over plants using a Preval CO_2_ pressurized sprayer [11, 12]. Sterile water was sprayed over control plants using the same method. A 10 μg/mL solution of Ptr ToxA was obtained from Dr. Timothy Friesen (USDA-ARS, North Dakota State University). A needleless syringe was used to inject this solution into leaf tissue [13], with sterile water injected as a control. This resulted in four different treatments for both Glenlea and Salamouni plants: (1) spray-inoculation with a suspension of *Ptr* spores (2) spray inoculation with water (3) needleless syringe injection with Ptr ToxA, and (4) needleless syringe injection with water. After treatments, plants were transferred to a humidity chamber and leaf sample were collected at 0, 8, and 16 h and flash-frozen in liquid nitrogen. Samples were transferred to a − 80 °C freezer.

### Extraction, sequencing, and analysis

RNA was extracted using the Ambion Purelink RNA extraction kit with Trizol reagent and treated with DNase. Samples were checked for the presence of 28S and 18S ribosomal subunits using gel electrophoresis and then sequenced at Iowa State University using Illumina HiSEQ 3000 (100 base pairs, single reads). As shown in Table 1, the resulting 48 RNA datasets are reported (Data Set 1) [14]. Figure S1 shows a flow chart that summarizes methods carried out during the experiment [15]. Data file 2 contains a spreadsheet with descriptions of the 48 RNA sequence datasets [15]. Read quality was assessed using the program FASTQC [16] and then trimmed using the program Btrim [17]. Mapping and assembly were carried out using the programs Hisat [18] and Htseq [19], respectively, aligning reads to the *Ptr* and wheat genomes (Data files 3 and 4, respectively) [15].

**Table 1:** Overview of data files/data sets

Label	Name of data file/data set	File types (file extension)	Data repository and identifier (DOI or accession number)
Figure S1	A flow chart summarizing methods used to carry out the tan spot of wheat experiment	image file (.png)	https://doi.org/10.6084/m9.figshare.8115980
Data set 1	NCBI Bioproject including all 48 sample accessions with associated fastq files	fastq (.fastq)	https://identifiers.org/ncbi/insdc.sra:SRP189899
Data file 2	Description of FASTQ files presented in Data set 1	spreadsheet (.xlsx)	https://doi.org/10.6084/m9.figshare.8115983
Data file 3	Count file after mapping to *Ptr* genome	count (.count)	https://doi.org/10.6084/m9.figshare.8115989
Data file 4	Count file after mapping to wheat genome	count (.count)	https://doi.org/10.6084/m9.figshare.8115992

## Limitations

We randomly selected two biological replicates from the six initially sampled of each treatment and time point in order to reduce the cost of sequencing. This limits how confidently we can label particular genes as differentially expressed and not the result of random variation.

## Data Availability

Raw FASTQ files were uploaded to the Sequence Read Archive (SRA) of the National Center for Biotechnology Information (NCBI) site. As Data Set 1, these files are available under the submission SUB5368694, Bioproject PRJNA529906 (https://identifiers.org/ncbi/insdc.sra:SRP189899) [14]. Figure S1 and Data files 2–4 were uploaded to Figshare (https://doi.org/10.6084/m9.figshare.8362244) [15].
